# Biochemical Characterization of Clinical Strains of *Staphylococcus* spp. and Their Sensitivity to Polyphenols-Rich Extracts from Pistachio (*Pistacia vera* L.)

**DOI:** 10.3390/pathogens7040082

**Published:** 2018-10-22

**Authors:** Erminia La Camera, Carlo Bisignano, Giuseppe Crisafi, Antonella Smeriglio, Marcella Denaro, Domenico Trombetta, Giuseppina Mandalari

**Affiliations:** 1 Department of Chemical, Biological, Pharmaceutical and Environmental Science, University of Messina, Viale SS. Annunziata, 98168 Messina, Italy; elacamera@unime.it (E.L.A.); gcrisafi@unime.it (G.C.); asmeriglio@unime.it (A.S.); denaromarcella.md@gmail.com (M.D.); dtrombetta@unime.it (D.T.); 2 Dipartimento di Scienze Biomediche, Odontoiatriche e delle Immagini Morfologiche e Funzionali, University of Messina, Via Consolare Valeria, 98125 Messina, Italy; cbisignano@unime.it

**Keywords:** *S. aureus*, pistachios, antimicrobial, MRSA, polyphenols

## Abstract

We characterized a number of clinical strains of *Staphylococcus* spp. and investigated their sensitivity against polyphenols-rich extracts from natural raw and roasted pistachios (NPRE and RPRE, respectively). Out of 31 clinical isolates of *Staphylococcus* spp., 23 were coagulase-positive and identified as *S. aureus*, of which 21 were MRSA. Polyphenols-rich extracts from natural pistachios and roasted pistachios were prepared: the total phenols content, expressed as gallic acid equivalent (GAE)/100 g fresh weight (FW), was higher in natural pistachios (359.04 ± 8.124 mg) than roasted pistachios (225.18 ± 5.055 mg). The higher total phenols content in natural pistachios also correlated to the higher free-radical scavenging activity found by DPPH assay: NPRE and RPRE showed IC_50_ values of 0.85 (C.L. 0.725–0.976 mg mL^−1^) and 1.15 (C.L. 0.920–1.275 mg mL^−1^), respectively. Both NPRE and RPRE were active against *S. aureus* 6538P and *Staph.* spp. clinical isolates, with RPRE being the most active (MIC values ranging between 31.25 and 2000 μg mL^−1^). The antimicrobial potential of pistachios could be used to identify novel treatments for *S. aureus* skin infections.

## 1. Introduction

*Staphylococcus aureus* and methicillin-resistant *S. aureus* (MRSA) are Gram-positive human pathogens responsible for a range of infections (skin, respiratory, and bone joint), endocarditis, bacteremia, and toxic shock syndrome [1]. *S. aureus* is implicated in a variety of biofilm-related infections, including implanted medical devices and wound-associated infections [2,3,4]. Biofilm formation has been reported in several human infections involving the oral cavity and the skin [5,6]. Biofilms are known to be resistant to conventional antibiotics and are therefore demanding for novel antibacterial compounds that can treat this community. Since several MRSA strains have become multi-drug resistant, novel treatments are needed to treat these widespread infections. We have reported the antibacterial activity of polyphenols-rich natural extracts, including almonds [7,8], *Citrus* plants [9], *Vitis vinifera* [10], *Olea europaea* L. [11], *Citrus bergamia* essential oil [12], and juice [13]. Phytochemicals previously identified from pistachios (*Pistacia vera* L.), including phytosterols, fatty acids, lutein, and tocopherols [14] have been involved with the health benefits associated with pistachio consumption. We have previously demonstrated that polyphenols from pistachios were bioaccessible in the upper gastrointestinal tract [15] and active against a range of Gram-positive bacteria [16]. Based on previous results, here we have characterized clinical isolates of *Staphylococcus* spp. in terms of presence of coagulase and lipase as well as biochemical API analysis and tested their sensitivity against polyphenol-rich extracts from pistachios. Furthermore, the extracts were tested on the production of bacterial biofilms in vitro.

## 2. Results

### 2.1. Polyphenols and Radical Scavenging Activity

A preventive phytochemical screening revealed a total phenols content (359.04 ± 8.124 and 225.18 mg ± 5.055 mg GAE/100 g FW for NP and RP, respectively). The higher total phenols content in NP was also correlated to the higher free-radical scavenging activity found by DPPH assay: NPRE and RPRE showed IC_50_ values of 0.85 (C.L. 0.725–0.976 mg mL^−1^) and 1.15 (C.L. 0.920–1.275 mg mL^−1^), respectively, which are well below those previously reported (1.30 to 2.39 mg mL^−1^) [17,18].

**Figure 1:**
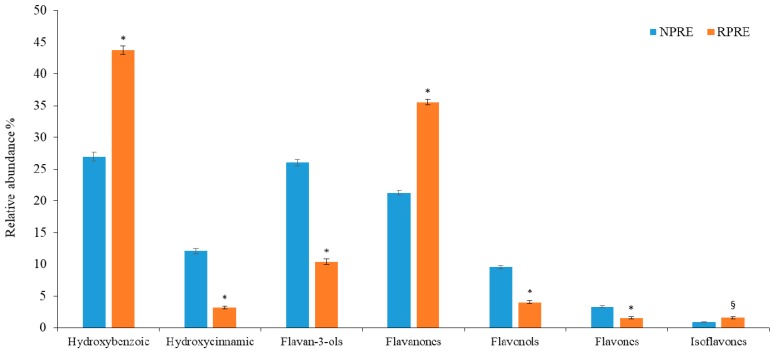
Polyphenol classes distribution within NPRE and RPRE. * *p* < 0.001; § *p* < 0.005.

These results were confirmed by RP-LC-DAD-FLU analysis, which highlighted a higher polyphenolic content of NP compared with RP (Table 1). Twenty-two polyphenols were identified and quantified, some for the first time, with respect to data previously reported [18]. Although the two extracts appeared similar in the phenolic acids and flavonoids content (39.02% and 60.98% in NPRE and 46.90 and 56.10% RPRE), a more in-depth analysis of the polyphenolic profile revealed substantial differences in the polyphenol classes contained (Figure 1). Despite the fact that hydroxybenzoic acids represent, among the phenolic acids, the most abundant class in both the extracts under study (26.95% and 43.75% in NPRE and RPRE, respectively), RPRE showed almost exclusively hydroxybenzoic acids (93.27% vs. 6.73% of hydroxycinnamic acids). Regarding flavonoids, NPRE was mainly composed of flavan-3-ols (42.66%), followed by flavanones (34.89%), flavonols (15.61%), flavones (5.40%), and isoflavones (1.45%). In contrast, RPRE mostly contained flavanones (66.96%), followed by flavan-3-ols (19.61%), flavonols (7.63%), isoflavones (2.94%), and flavones (2.87%).

### 2.2. Phenotypic Identification of Staphylococcus Strains

The phenotypic characterization of the clinical *Staphylococcus* strains is reported in Table 2. Out of the 31 clinical isolates, 23 were coagulase-positive and identified as *S. aureus*, two strains were coagulase and lipase negative and identified as *S. epidermidis*, two strains were coagulase and lipase negative and identified as *S. lugdunensis*, two strains were coagulase and lipase negative and identified as *S. hominis*, one strain was coagulase and lipase negative and identified as *S. xylosus,* and one unidentified strain of *Staphylococcus* was coagulase and lipase negative. Interestingly, 21 out 23 *S. aureus* strains were MRSA. Ten (10) out of 23 *S. aureus* strains did not produce lipase: 6 out of 16 strains isolated from knee prosthesis produced lipase and 4 out of 7 from hip prosthesis produced lipase. 

### 2.3. Antimicrobial Activity of Pistachio Extracts

The MIC and MBC values of NPRE and RPRE are shown in Table 3. Negative controls indicated absence of inhibition (data not shown).

Both NP and RP polyphenolic extracts were active against the Gram-positive bacteria, with RPRE (activity against ATCC strain and 17 out of 31 clinical isolates) overall being more effective than NPRE (activity against ATCC strain and 25 out of 31 clinical isolates). The activity was bacteriostatic rather than bactericidal. No effect on biofilm production was detected using the pistachio extracts.

## 3. Discussion

The present work further validates the findings of antimicrobial potential of polyphenols-rich extracts against Gram-positive bacteria, both standard and clinical isolates. We have demonstrated that extracts from pistachios were partially active against clinical strains of *Staphylococcus* spp., some of which were multi-drug resistant. Pathogenicity of *S. aureus* is attributed to a wide range of virulence factors, including extracellular protease, lipase, and superoxide dismutase. The increased prevalence of multidrug resistance *S. aureus* poses a serious risk to worldwide public health, and novel treatment strategies are needed to address this concern. Over the last decade, MRSA strains have become one of the main causes of mortality amongst hospital-acquired infectious diseases [19,20]. *S. aureus* 6538P was the most sensitive strain, with complete inhibition achieved with a concentration of 125 and 31.25 μg mL^−1^ of NPRE and RPRE, respectively. Overall, we have found RPRE was more effective than NPRE. This trend, which cannot be explained by the total amount of polyphenols present in the two extracts, or by their antioxidant potential, could be attributed to the qualitative composition of the two extracts (Figure 1). In agreement with our previous investigation [15], the concentration of gallic acid and eryodictiol-7-O-glucoside was higher in RP than NP. Lee et al. [21] have recently reported on the antibacterial activity of a multifunctional nanoparticle containing gallic acid against methicillin resistant *S. aureus* strains: the bactericidal activity of functionalized nanoparticles containing gallic acid was increased compared to the non-functionalized nanoparticles, with high selectivity for MRSA strains. Extracts from phenolic blueberry and blackberry pomace rich in phenolic acids, mainly protocathecuic, cumaric, vanillic, caffeic, and gallic acids, were able to inhibit the growth of vegetative MRSA in vitro and MRSA biofilm formation on plastic surface [22]. A recent investigation [23] reported on the protective role played by eriodyctiol against *S. aureus* induced lung cell injury by inhibiting alpha-hemolysin expression.

The differences between the two extracts, possibly due to the roasting procedure, may affect their biological activity. It is known that each class of polyphenols is characterized by an activity closely related to its chemical structure, due mainly to hydroxyl groups linked to phenolic structures and their degree of glycosylation [24,25]. Amongst flavanols, catechin was nearly 3 times higher in RPRE compared with NPRE, whereas hydroxybenzoic acids and flavanones were significantly higher in roasted pistachios (Figure 1). From our preliminary investigations, it was observed that NPRE was richer in polyphenols and consequently had greater antioxidant activity compared to RPRE. The total phenols values were higher than those previously observed, which substantially vary with regard to the pistachio kernels, ranging from 165 ± 8.00 to 347 ± 34.00 mg GAE/100 g [17,18]. Natural raw pistachios were found more active than roasted salted pistachios in our recent in vitro and in vivo studies [26,27].

Galloyl flavan-3-ols such as (-)-epicatechin gallate and catechins are effective against MRSA strains [28], whereas (-)-epicatechin gallate sensitises MRSA strains to β-lactam antibiotics [29,30]. The use of pistachio polyphenols in combination with traditional or antibiotics could identify new mechanisms of synergism and modulate properties of antibiotic resistance. This could aim to the development of novel topical agents for the treatment of *S. aureus* skin infections as well as for topical formulations.

In conclusion, the results of the present study demonstrated that polyphenols from pistachios are effective against ATCC strains of *S. aureus* and clinical strains of *Staph.* spp. Further studies are needed to establish possible synergistic effect with antibiotics in order to develop novel chemotherapic agents for the treatment of *S. aureus* infections.

## 4. Materials and Methods

### 4.1. Pistachio Extracts

Californian natural shelled (NP) and roasted (RP) pistachio kernels were kindly provided by Di Bartolo S.r.l., Calatabiano (Italy).

NPs and RPs were ground to fine powder by an analytical blade mill (IKA® A11), under liquid nitrogen, in order to block enzymatic activities and preserve organoleptic and nutritional properties. Polyphenols-rich extracts of NP (NPRE) and RP (RPRE) were obtained following the method reported by Mandalari et al*.* [15].

### 4.2. Total Phenols

The total phenol content of NPRE and RPRE was determined colorimetrically using the Folin-Ciocalteu assay as previously described by Smeriglio et al. [31] and expressed as mg of gallic acid equivalents (GAE)/100 g of NP and RP FW. Results represent the average ± standard deviation (SD) of three independent experiments (*n* = 3).

### 4.3. Radical Scavenging Activity

The anti-radical activity of NPRE and RPRE was determined using the stable 2,2-diphenyl-1-picrylhydrazyl radical (DPPH^•^) according to Smeriglio et al*.* [32]. The inhibition (%) of radical activity was calculated using the following equation (Equation (1)):

(1) $$\mathrm{Inhibition}\;\left(\%\right)=\;\frac{A_{0}-A_{s}}{A_{0}}\;\times 100$$

in which A_0_ is the absorbance of the control and A_s_ is the absorbance of the sample after 20 min incubation. Results were expressed as half-inhibitory concentration (IC_50_) calculating the confidence limit (C.L.) at 95%.

### 4.4. Polyphenolic Profile

Determination of polyphenol profile was carried out by LC-DAD-FLU analysis according to Bisignano et al. [33]. Polyphenols were allowed by comparing peak’s UV-Vis spectra and retention times with those of commercially available reference compounds (purity ≥ 99%. Extrasynthese, Genay, France) and using, for quantitative analysis, external standard calibration curves (concentration range 1–50 μg/mL).

The results were expressed as milligrams of each compound/100 g of NP and RP FW and represent the average ± standard deviation (SD) of three independent experiments (*n* = 3).

### 4.5. Microbial Strains, Culture Conditions, and Phenotypic Characterization

The following strains, obtained from the University of Messina’s in-house culture collection (Messina, Italy), were used: *Staphylococcus aureus* ATCC 6538P, and 31 clinical isolates of *S.* spp. obtained from swabs of patients with an orthopedic infection. Out of the 31 clinical isolates, 16 were obtained from a knee prosthesis or surgical wound, 7 from hip prosthesis, and 8 from other orthopedic sites. All the swabs were cultivated on 5% sheep blood agar plates (Oxoid, Basingstoke, UK) and incubated for 24–48 h at 37 °C under aerophilic condition. Strains were identified by conventional methods, presumptively by colony morphology, Gram staining, selective isolation on Baird Parker agar base with egg yolk tellurite emulsion (Oxoid), catalase, and coagulase test (Staphylase Test, Oxoid), and stored in BHI containing 10% glycerol (vol/vol) at −70 °C. All isolates were revitalized on 5% sheep blood agar and tested for species identification phenotypically by the analytical profiling index using an API identification system (Api Staph, BioMerieux, Marcy-l’Étoile, France).

The principle of the API system is to generate an identification code from individual miniaturized biochemical reactions, each producing either a positive or negative result. The composite of the binary results is converted into a numerical profile, which is then entered into a database for the generation of the identification of the microorganism.

### 4.6. Lipase Activity

Lipase activity was monitored on MHT plates containing Muller-Hinton agar medium, 1% Tween 60, and 0.01% CaCl_2_. Ten (10) µL of overnight TSB cultures diluted to 10^5^ cells were inoculated by spotting on the plate surfaces and incubated at 37 °C for 72 h. The colonies were observed daily under a stereo microscope (Stereo eighty, Swift Instr. International S.A., Boulder, CO, USA). All determinations were performed in duplicate.

### 4.7. Susceptibility Studies

The minimum inhibitory concentration (MIC) and the minimum bactericidal concentration (MBC) of NPRE and RPRE were determined using a broth microdilution method in 96-well round- bottomed polystyrene microtiter plates according to Clinical and Laboratory standards Institute [34]. Briefly, the assay was executed in Mueller–Hinton broth (MHB) using overnight cultures. The employed strain inoculum was 1–5 × 10^5^ CFU mL^−1^. Stock solutions of each extract in DMSO (100 mg mL^−1^) were diluted in MHB to give serial 2-fold dilutions, which were added to each well in order to obtain final concentrations ranging from 2000 to 3.9 µg mL^−1^. The final concentration of DMSO in the assay did not exceed 1%. MHB and DMSO (1%) were used as negative controls. Plates were incubated at 37 °C for 24 h. The MIC values were defined as the lowest extract concentrations showing no bacterial growth after the incubation time. MBCs were determined by seeding 20 µL from all clear MIC wells onto Mueller-Hinton agar (MHA, Oxoid) plates. The MBC was defined as the lowest extract concentration, which killed 99.9% of the final inocula after 24 h incubation at 37 °C. All assays were done in triplicate. Positive and negative controls with antibiotics and solvent (DMSO) were included in each assay.

### 4.8. Effect on Biofilm Formation

The effect of different concentrations of NPRE and RPRE (ranging from 1/2 MIC to 1/16 × MIC) on biofilm forming ability was tested using polystyrene flat-bottomed microtiter plates (Costar) [35,36]. Briefly, bacterial cultures were grown overnight in Tryptic Soy Broth (TSB) + 1% glucose, diluted in the same medium to 1–5 × 10^6^ CFU mL^−1^, and dispensed into each well of microtiter plate (100 µL) in presence of 100 µL twofold serial dilution of each extract. The bacterial strains in the absence of antibacterial agents and TSB with DMSO were included as controls. After 24 h of incubation a 37 °C, biofilm inhibition was quantified. The supernatants were decanted, and cells removed by PBS washing (pH 7.2). The biofilm was fixed with methanol for 15 min and air dried at temperature, then stained with 0.1% (*w*/*v*) safranin (Sigma) for 5 min and rinsed thoroughly with water. To quantify biofilm formation, 200 µL of 30% acetic acid were added to each well for 30 min. The absorbance was determined by using the spectrophotometer EIA reader at 492 nm. The biofilm reduction was calculated as follows:

100 − (mean OD492 of treated well/mean OD492 of control well) × 100

Each assay was performed in duplicate and repeated at least three times.

### 4.9. Statistical Analysis

Results were expressed as mean ± standard deviation (S.D.) of three independent experiments in triplicate (*n* = 3) and analyzed by one-way analysis of variance (ANOVA). The significance of the difference was assayed by using Tukey’s test for each paired experiment using a SigmaPlot 12.0 software. Statistical significance was considered at *p* < 0.05.

## Figures and Tables

**Table 1:** Polyphenolic profile of natural and roasted pistachios. Value are expressed as mg/100 g FW and represent average (± SD) of three independent experiments (*n* = 3).

Compound	NPRE	RPRE
*Hydroxybenzoic acids*		
Gallic acid	0.99 ± 0.035	1.77 ± 0.055
Protocathecuic acid	1.01 ± 0.042	1.08 ± 0.047
Hydroxybenzoic acid	0.17 ±0.011	0.19 ± 0.008
Vanillic acid	0.02 ± 0.001	-
*Hydroxycinnamic acids*		
Chlorogenic acid	0.15 ± 0.011	0.19 ± 0.010
Caffeic acid	0.71 ± 0.032	0.01 ± 0.001
Cumaric acid	0.12 ± 0.010	0.02 ± 0.001
*Flavanones*		
Eryodictiol	0.23 ± 0.012	0.21 ± 0.013
Eryodictiol-7-*O*-glucoside	1.34 ± 0.088	2.19 ± 0.074
Naringenin	0.05 ± 0.002	0.03 ± 0.002
Naringin	0.10 ± 0.004	0.04 ± 0.001
*Flavonols*		
Kaempferol-3-*O*-rutinoside	0.05 ± 0.002	0.04 ± 0.002
Quercetin	0.18 ± 0.010	0.07 ± 0.003
Quercetin-3-*O*-rutinoside	0.30 ± 0.016	0.13 ± 0.008
Quercetin-3-*O*-glucoside	0.24 ± 0.012	0.04 ± 0.002
*Flavones*		
Amentoflavone	0.17 ± 0.007	0.05 ± 0.003
Luteolin	0.04 ± 0.002	0.04 ± 0.002
Apigenin	0.06 ± 0.001	0.01 ± 0.001
*Isoflavones*		
Daidzein	0.06 ± 0.002	0.10 ± 0.005
Genistein	0.01 ± 0.001	0.01 ± 0.004
*Flavanols*		
Epicatechin	0.07 ± 0.002	0.04 ± 0.002
Catechin	2.04 ± 0.080	0.69 ± 0.035
*Total amount*	8.11	6.95

**Table 2:** Phenotypic characterization of *Staphylococcus* strains. Numbers from 1 to 31 indicate clinical strains. + = positive; − = negative. OOS = other orthopedic site.

Strain	Origin	Coagulase	Lipase	API System
1	knee	+	−	*S. aureus*
2	hip	+	+	*S. aureus*
3	knee	+	+	*S. aureus*
4	OOS	+	+	*S. aureus*
5	knee	−	−	*S. sp.*
6	OOS	−	−	*S. xylosus*
7	hip	+	−	*S. aureus*
8	OOS	+	+	*S. aureus*
9	knee	+	+	*S. aureus*
10	knee	+	−	*S. aureus*
11	hip	+	+	*S. aureus*
12	knee	−	−	*S. epidermidis*
13	OOS	−	−	*S. lugdunensis*
14	knee	−	−	*S. epidermidis*
15	hip	+	−	*S. aureus*
16	OOS	+	+	*S. aureus*
17	OOS	+	+	*S. aureus*
18	knee	+	+	*S. aureus*
19	knee	+	−	*S. aureus*
20	knee	+	−	*S. aureus*
21	knee	+	−	*S. aureus*
22	knee	−	+	*S. hominis*
23	hip	+	−	*S. aureus*
24	knee	−	+	*S. hominis*
25	knee	+	+	*S. aureus*
26	OOS	+	+	*S. aureus*
27	hip	+	+	*S. aureus*
28	OOS	+	+	*S. aureus*
29	hip	−	−	*S. lugdunensis*
30	knee	+	−	*S. aureus*
31	knee	+	−	*S. aureus*
ATCC 6538P		+	+	*S. aureus*

**Table 3:** MICs (μg mL^−1^) of pistachios against *Staphylococcus* strains.

Strain	NPRE	RPRE
**1**	2000	2000
**2**	>2000	2000
**3**	>2000	2000
**4**	250	500
**5**	>2000	>2000
**6**	2000	2000
**7**	>2000	1000
**8**	>2000	2000
**9**	2000	2000
**10**	2000	2000
**11**	>2000	1000
**12**	2000	2000
**13**	2000	2000
**14**	62.5	500
**15**	1000	1000
**16**	>2000	>2000
**17**	>2000	1000
**18**	2000	2000
**19**	2000	2000
**20**	2000	2000
**21**	>2000	2000
**22**	>2000	>2000
**23**	2000	2000
**24**	2000	2000
**25**	>2000	2000
**26**	>2000	2000
**27**	>2000	>2000
**28**	>2000	>2000
**29**	>2000	>2000
**30**	1000	1000
**31**	2000	2000
**ATCC 6538P**	125	31.25
